# Computational Issues of Quantum Heat Engines with Non-Harmonic Working Medium

**DOI:** 10.3390/e26050359

**Published:** 2024-04-25

**Authors:** Andrea R. Insinga, Bjarne Andresen, Peter Salamon

**Affiliations:** 1 Department of Energy Conversion and Storage, Technical University of Denmark, DK-2800 Kgs. Lyngby, Denmark; 2 Niels Bohr Institute, University of Copenhagen, Blegdamsvej 17, DK-2100 Copenhagen, Denmark; andresen@nbi.ku.dk; 3 Department of Mathematics and Statistics, San Diego State University, San Diego, CA 92182-7720, USA; salamon@sdsu.edu

**Keywords:** Otto cycle, quantum engine, quartic oscillator, density matrix, Schrödinger picture

## Abstract

In this work, we lay the foundations for computing the behavior of a quantum heat engine whose working medium consists of an ensemble of non-harmonic quantum oscillators. In order to enable this analysis, we develop a method based on the Schrödinger picture. We investigate different possible choices on the basis of expanding the density operator, as it is crucial to select a basis that will expedite the numerical integration of the time-evolution equation without compromising the accuracy of the computed results. For this purpose, we developed an estimation technique that allows us to quantify the error that is unavoidably introduced when time-evolving the density matrix expansion over a finite-dimensional basis. Using this and other ways of evaluating a specific choice of basis, we arrive at the conclusion that the basis of eigenstates of a harmonic Hamiltonian leads to the best computational performance. Additionally, we present a method to quantify and reduce the error that is introduced when extracting relevant physical information about the ensemble of oscillators. The techniques presented here are specific to quantum heat cycles; the coexistence within a cycle of time-dependent Hamiltonian and coupling with a thermal reservoir are particularly complex to handle for the non-harmonic case. The present investigation is paving the way for numerical analysis of non-harmonic quantum heat machines.

## 1. Introduction

We discuss the computational methods that we have developed for the simulation of the behavior of a quantum heat engine whose working fluid is an ensemble of quartic oscillators. The type of quantum systems composing the ensemble that acts as the working medium is one of the distinguishing aspects between the many different works published within this area. Various possibilities have been investigated, such as ensembles of photons [1], of individual spins [2], quadrupolar nuclear spins [3], fermions bound to a linear chain with *N* sites [4], or oscillators [5].

The study of decoherence, dephasing, and dissipation phenomena requires simulation of the time evolution of the state of a statistical ensemble of quantum systems. The state of the ensemble can be represented through the use of the density operator formalism. Evolution in the presence of dissipation is represented by a non-unitary equation of motion and therefore cannot be reduced to the evolution of several independent single-particle wave functions without including the dissipation mechanism in some way, as is conducted in, e.g., the quantum jump formalism [6]. The numerical integration of the evolution equation for the density matrix is not problematic for an ensemble of finite-dimensional systems, such as an ensemble of pairs of interacting two-level spin-systems, extensively studied by Kosloff and Feldmann [7,8,9,10] and also demonstrated experimentally [11]. When the dimension of the Hilbert space of the system is infinite, this procedure is not straightforward since it is necessary to reduce the dimensionality, and an additional source of approximation is thus introduced.

However, in some particular cases, the analytical properties of the governing equations lead to *canonical invariance*. When this condition is verified, the state of the ensemble of systems can be represented by a finite number of parameters. This property significantly reduces the computational cost of the simulations, since it is possible to avoid the integration of the time-evolution equation for the matrix expansion of the full density operator. The harmonic oscillator [12,13,14,15] is the prototypical example of an infinite-dimensional system exhibiting canonical invariance. Many studies on this topic [16,17,18,19] adopt the Heisenberg formalism, where the state of the ensemble is represented by the expectation values of a small set of appropriately chosen operators using the dynamical symmetry group of the problem.

In this work, we address the problem of simulating the dissipative dynamics of an ensemble of non-harmonic oscillators. As a model for non-harmonicity, we choose a potential that includes not only the usual quadratic term but also a small quartic term. This term breaks the symmetry properties of the harmonic case, thus negating canonical invariance. For this reason, it is necessary to analyze the system in the Schrödinger picture, i.e., to compute the evolution of the density matrix. Naturally, since the dimension of the underlying Hilbert space is infinite, it is also necessary to perform some reduction in order to obtain a finite set of numerically integrable equations.

We put particular effort into the selection of a computationally efficient basis on which we expand the operators. The work is inspired by previous works on thermodynamic cycles of quantum systems out of equilibrium, especially the studies by Rezek et al., such as [16,17,18]. For this reason, we also have to consider the problem of how to determine the *limit cycle* for the particular choice of parameters. Finally, we employ different methods in order to verify whether the result produced by a simulation is reliable and satisfies the required physical properties.

The algorithm that we constructed for simulating the quartic heat engine is sufficiently efficient and accurate to be used for the thermodynamic study of this system. The purpose of the thermodynamic analysis may vary widely. Many previous works set themselves in the area of finite-time thermodynamics and adopt an optimization perspective. When a refrigeration cycle is chosen, the objective could be the optimization of the cooling power [7,20] or the refrigeration toward zero temperature [17]. For an engine-like cycle, the objective is often the optimization of the power output [10,16]. In the latter reference in particular, it is investigated how the dephasing phenomenon can be exploited to suppress quantum friction and increase the power output. We choose an engine-like cycle and focus on the optimization of the power output. We will report our results from this thermodynamic analysis in an upcoming paper. Here, we focus on the numerical techniques that can guarantee the reliability of our conclusions, and whereas the non-unitary evolution of ensembles of non-harmonic quantum oscillators has been investigated before [21,22,23,24], here we analyze the case of an ensemble undergoing an Otto thermodynamic cycle. The computational and error-estimation techniques discussed in the present are specific to the analysis of quantum heat cycles, due to the co-existence of dissipative dynamics and time-dependent Hamiltonian within the same cycle. As will be discussed in detail, this situation involves specific challenges and nuances that are addressed in the present work.

We believe that the methods explored in the present work may be used for the study of a variety of systems with appropriate modifications since they do not rely on special analytical properties of the chosen system.

## 2. System

### 2.1. Notation

In this paper, we employ the bra-ket notation, i.e., elements of the Hilbert space are denoted by kets, as in |n〉, and elements of the dual space by bras, as in 〈n|. We denote linear operators with the ^ symbol, as in $\hat{X}$. The expectation value of an operator $\hat{X}$ is denoted by $\langle \hat{X}\rangle$ or simply by *X*. Super-operators, i.e., linear transformations acting on the vector space of operators, are denoted with the calligraphic font, as in L. If ${\{|n\rangle \}}_{n}$ is a basis, $\langle n|\hat{X}|m\rangle$ denotes one entry of the matrix expansion of $\hat{X}$ over the basis. The trace of an operator $\hat{X}$ is denoted by $\mathrm{Trace}\left[\hat{X}\right]$:

(1) $$\mathrm{Trace}\left[\hat{X}\right]=\sum_{n}\langle n|\hat{X}|n\rangle$$

### 2.2. Hamiltonian

Each of the quantum systems that constitute the working medium of the engine is governed by the Hamiltonian

(2) $${\hat{H}}_{\alpha }=\frac{1}{2m}{\hat{P}}^{2}+\frac{1}{2}m\omega^{2}\left({\hat{Q}}^{2}+\alpha {\hat{Q}}^{4}\right)$$

where $\hat{Q}$ denotes the position operator, $\hat{P}$ the momentum operator, ω the frequency of the oscillator, *m* its mass, and α the strength of the quartic term. The cycle of operation of the engine is composed of two adiabatic and two isochoric processes and is the quantum analog of the classical Otto cycle, and whereas *m* and α are assumed to remain constant throughout the cycle, ω is time dependent during the adiabatic processes.

### 2.3. Adiabatic Evolution

Because of the variation in the scalar parameter ω, the Hamiltonian is explicitly time dependent during the adiabatic process, and therefore the energy 〈H〉 of the system is not constant. The energy variation is interpreted as work exchanged between the working medium and the surroundings. It is important to stress that for a finite-time process, the quantum adiabatic theorem predicts that not only the eigen energies of the Hamiltonian will be time dependant, but also the probabilities of occupation of the energy levels. In the context of the quantum adiabatic theorem, the term “adiabatic” means that the time evolution is quasistatic, i.e., the Hamiltonian is varied sufficiently slowly as not to alter the probability distribution among the energy levels. Elsewhere in the manuscript, we use the term “adiabatic evolution” with the conventional meaning adopted in thermodynamics, i.e., a process during which the system is thermally insulated. When the evolution is not quasistatic, there is a contribution to the energy exchange between system and surrounding that is due to the variation in the probability distribution among the energy levels. This energy exchange term is interpreted as *quantum friction* [9,10].

The parameter ω varies from an initial value to a final value in a specified time. A time law for its time dependence, a driving protocol, has to be selected among all the possible choices of ω(t). Three possibilities are frequently considered in the literature: constant rate of variation $\dot{\omega }$, constant relative rate of variation $\lambda =\dot{\omega }/\omega$ (also known as the *nonadiabatic parameter* [16]), and constant dimensionless adiabatic parameter [17]$\mu =\dot{\omega }/\omega^{2}$. The particular choice of the time dependence of ω is important when analytical methods are employed for the solution of the relevant equations. In the harmonic case, i.e., α=0, the choice for which μ is time-independent leads to an analytically integrable set of equations; the details can be found in [17]. In the present study, however, we will focus on numerical techniques and therefore the particular choice of driving protocol does not affect the mathematical treatment of the problem. We decided to follow the choice of the seminal work of Rezek [16] and select the time law for which λ is constant such that ω(t)=exp(λt). It is important to stress that all the methods and results presented in this paper would be completely equivalent if a different driving protocol had been chosen.

The nonadiabatic parameter is not dependent on the mass *m* of the oscillator and it completely characterizes the dynamics during the adiabatic process. The two opposite limits, a quasi-static and a sudden process, are associated, respectively, with the limits $\lambda \to 0^{+}$ and λ→+∞.

The state of the system is represented by the density operator $\hat{\rho }$ which evolves according to the analog of the Schrödinger equation in the density operator formalism, called the *Liouville-von Neumann equation*,

(3) $$\frac{d}{dt}\hat{\rho }\left(t\right)=-\frac{i}{ћ}\left[\hat{H}\left(t\right),\hat{\rho }\left(t\right)\right]=L_{H}\left(\hat{\rho }\right)$$

The symbol $L_{H}$ denotes the unitary Liouville super-operator [6].

### 2.4. Isochoric Evolution

During the isochoric processes, the Hamiltonian operator does not explicitly depend on time. However, the energy 〈H〉 of the ensemble is not constant. The energy variation is caused by the interaction of the working fluid with the thermal reservoirs and is included as an additional non-unitary term in the Liouville-von Neumann equation

(4) $$\frac{d}{dt}\hat{\rho }\left(t\right)=L_{H}\left(\hat{\rho }\right)+L_{D}\left(\hat{\rho }\right)$$

Here, the symbol $L_{D}$ denotes the Lindblad dissipative super-operator [6,25], defined as:

(5) $$L_{D}\left(\hat{\rho }\right)=\sum_{\sigma =↑↓}k_{\sigma }\left({\hat{A}}_{\sigma }^{†}\hat{\rho }\left(t\right){\hat{A}}_{\sigma }-\frac{1}{2}\left\{{\hat{A}}_{\sigma }^{†}{\hat{A}}_{\sigma },\hat{\rho }\left(t\right)\right\}\right)$$

Equation (4) is also referred to as the Lindblad equation. The two different terms, labeled by the index σ, are associated with two different transition channels which correspond to the two Lindblad operators, ${\hat{A}}_{↑}$ and ${\hat{A}}_{↓}$, and their transition rates $k_{↑}$ and $k_{↓}$. Since the energy levels are equally spaced for the harmonic case, it is natural to select as Lindblad operators the harmonic creation and annihilation operators, given by

(6) $${\hat{A}}_{↓}=\hat{a}=\frac{1}{\sqrt{2}}\left(\left(\frac{\sqrt{m\omega }}{\sqrt{ћ}}\right)\hat{Q}+i\left(\frac{1}{\sqrt{m\omega ћ}}\right)\hat{P}\right)$$

(7) $${\hat{A}}_{↑}={\hat{a}}^{†}=\frac{1}{\sqrt{2}}\left(\left(\frac{\sqrt{m\omega }}{\sqrt{ћ}}\right)\hat{Q}-i\left(\frac{1}{\sqrt{m\omega ћ}}\right)\hat{P}\right)$$

This choice automatically guarantees that the detailed balance condition is fulfilled as long as the ratio between the transition rates respects the property $\frac{k_{↑}}{k_{↓}}=\exp\left(-\beta ћ\omega \right)$, where β is the inverse temperature of the heat reservoir. For the non-harmonic case, the energy levels are not equally spaced. For this reason, an exact treatment of the dissipation would require an infinite number of transition channels. Fortunately, previous works [26,27] on the dissipation with non-harmonic working fluids point out that an approximate treatment is possible with two transition channels even if the position dependence of the Lindblad operators is allowed to be a more general function of $\hat{Q}$, not necessarily linear.

The value of the inverse temperature β of the heat reservoir determines the ratio between the transition rates. However, it is possible to arbitrarily fix the value of the difference Γ between them, $\Gamma =k_{↓}-k_{↑}$. The parameter Γ is interpreted as the heat conductance between the system and the thermal reservoir.

### 2.5. The Otto Cycle

The different processes combined in the following order give one complete thermodynamic cycle:

- *Hot Isochore*—The system, whose frequency is equal to $\omega_{H}$, is coupled to the hot heat reservoir whose inverse temperature is $\beta_{H}$. The heat conductance is equal to $\Gamma_{H}$.
- *Expansion Adiabat*—The frequency of the system changes from $\omega_{H}$ to $\omega_{C}$.
- *Cold Isochore*—The system, whose frequency is equal to $\omega_{C}$, is coupled to the cold heat reservoir whose inverse temperature is $\beta_{C}$. The heat conductance is equal to $\Gamma_{C}$.
- *Compression Adiabat*—The frequency of the system changes from $\omega_{C}$ to $\omega_{H}$.

The durations of the four processes are, respectively, denoted by $\tau_{H}$, $\tau_{HC}$, $\tau_{C}$, and $\tau_{CH}$. The duration of a complete cycle is thus $\tau =\tau_{H}+\tau_{HC}+\tau_{C}+\tau_{CH}$. For an engine-like cycle, the following relations need to be satisfied: $\beta_{C}>\beta_{H}$ and $\omega_{C}<\omega_{H}$.

### 2.6. Limit Cycle

It is possible to write a formal solution to the evolution equations for the density operator $\hat{\rho }$. All the equations involved are of the form $\frac{d}{dt}\hat{\rho }=L\left(\hat{\rho }\right)$ and all the solutions then can be written as $\hat{\rho }\left(t\right)=U_{t}\left({\hat{\rho }}_{0}\right)$, where $U_{t}$ is the correct time evolution super-operator and ${\hat{\rho }}_{0}$ is an arbitrary initial state.

We are interested only in the *limit cycle* solutions, the solutions for which, at the initial and final instants of the thermodynamic cycle, the state of the system is represented by the same density operator. We denote a solution belonging to this category with the symbol ${\hat{\rho }}^{\infty }$. We therefore have to impose the additional invariance condition $U_{\tau }\left({\hat{\rho }}^{\infty }\right)={\hat{\rho }}^{\infty }$.

Similarly to the procedure described in Ref. [28], the limit cycle could be computed by solving with respect to ${\hat{\rho }}^{\infty }$ the linear equation $\left(U_{\tau }-I\right){\hat{\rho }}^{\infty }=\hat{0}$, where I denotes the identity super-operator, and $\hat{0}$ the null operator. Due to the large number of variables required for the non-harmonic problem, this direct inversion seems to be unfeasible. However, a useful property may help, namely that in many cases the limit cycle is *attractive*, i.e., *stable*. This property can be expressed by the equation

(8) $$\lim_{N\to \infty }U_{\tau }^{N}\left({\hat{\rho }}_{0}\right)={\hat{\rho }}^{\infty }\;;\;\forall \;{\hat{\rho }}_{0}$$

The implication, convenient from a computational point of view, is that a repeated application of the full cycle evolution super-operator $U_{\tau }$ will eventually lead to the limit cycle solution ${\hat{\rho }}^{\infty }$, regardless of the choice of the initial state ${\hat{\rho }}_{0}$.

## 3. The Evolution Algorithm

### 3.1. Expansion Equations

In some cases, the most studied being the harmonic case, there exists a useful property that allows one to represent the state of the system with a finite number of scalar parameters [16]. A finite set of Hermitian operators ${\hat{X}}_{k}$ has to be found so that the two following properties are fulfilled:

- The set forms a *Lie algebra*, i.e., is closed under the application of the commutator between any pair of operators:
  (9) $$\left[i{\hat{X}}_{h},i{\hat{X}}_{j}\right]=i\sum_{k=1}^{K}\Gamma_{hjk}{\hat{X}}_{k},\;\mathrm{with}\;\Gamma_{hjk}\in R\;\forall \;h,j,k$$
- The algebra is closed under the application of $L_{H}$ and $L_{D}$. For $L_{H}$, this requirement means that it is possible to write the Hamiltonian operator $\hat{H}$ as a linear combination with real coefficients of the operators in the set,
  (10) $$\hat{H}=\sum_{k=1}^{K}c_{k}{\hat{X}}_{k},\;with\;c_{k}\in R\;\forall \;k$$
  For $L_{D}$, the closure requirement leads to additional conditions on the Lindblad operators ${\hat{A}}_{↓}$ and ${\hat{A}}_{↑}$ [16].

When these conditions are met, *canonical invariance* holds and it is possible to represent the state of the system with a finite number of parameters for the whole evolution [16]. This representation is different for the Schrödinger and Heisenberg formalisms.

In the *Schrödinger formalism*, the density operator $\hat{\rho }$ is time dependent. Because of canonical invariance, an initial state of the form $\hat{\rho }=\frac{1}{Z}\exp\left(\sum_{k}\beta_{k}{\hat{X}}_{k}\right)$ remains in the same form for all previous and future instants of the evolution, i.e., $\hat{\rho }\left(t\right)=\frac{1}{Z(t)}\exp\left(\sum_{k}\beta_{k}\left(t\right){\hat{X}}_{k}\right)$. The value of the parameters $\beta_{k}$ will then completely determine the state of the system at any given moment.

In the *Heisenberg formalism*, the set of operators is time dependent. Because of canonical invariance, the equations of motion of the operators forming the Lie algebra assume the closed form

(11) $$\frac{d}{dt}{\hat{X}}_{k}=\sum_{j=1}^{K}a_{kj}{\hat{X}}_{j},\;for\;k=1,\cdots ,K$$

The expectation values of these operators obey the same Equation (11). It is then possible to describe the dynamics of the system with the time dependence of the expectation values $\langle {\hat{X}}_{k}\rangle \left(t\right)$. In this way, it is possible to calculate the time evolution of the energy together with those observables unavoidably coupled to the Hamiltonian operator.

Since Lie algebras are vector spaces, the choice of a basis of operators, such as the set $\left\{{\hat{X}}_{k}\right\}$, is not unique. It is possible to construct alternative sets by choosing linear combinations of the operators in the original set. For the harmonic case, one possible choice [16] is the Hamiltonian $\hat{H}$, the Lagrangian $\hat{L}$, and the correlation operator, $\hat{D}=\hat{Q}\hat{P}+\hat{P}\hat{Q}$. Another possibility [18] is the set $\left\{{\hat{Q}}^{2},{\hat{P}}^{2},\hat{D}\right\}$. In both these cases, the identity operator $\hat{1}$ has to be included when considering the dissipative evolution.

Because of the ${\hat{Q}}^{4}$ term present in Equation (2), it is not possible to construct a finite-dimensional Lie algebra that is closed under the application of $L_{H}$ and $L_{D}$. In fact, it can be shown that as long as the position-dependent potential includes terms of power greater than 2, it is not possible to construct a finite-dimensional Lie algebra which is closed with respect to $L_{H}$ [29]. Therefore, the conditions for canonical invariance are not satisfied for the non-harmonic case, and it is thus necessary to consider a general density operator. For this reason, we decided to expand $\hat{\rho }$ on a basis, {|n〉}, and numerically integrate the resulting coupled equations of motion for all the matrix elements, $\langle n|\hat{\rho }|m\rangle$, of the expansion

(12) $$\frac{d}{dt}\langle n|\hat{\rho }|m\rangle =\langle n|L_{H}\left(\hat{\rho }\right)|m\rangle +\langle n|L_{D}\left(\hat{\rho }\right)|m\rangle$$

For the calculation, it is necessary to compute also the matrix expansion in the same basis of all the operators appearing in the unitary and dissipative super-operators, i.e., $\hat{P}$, $\hat{Q}$, ${\hat{P}}^{2}$, ${\hat{Q}}^{2}$, and ${\hat{Q}}^{4}$.

### 3.2. Choice of Basis

The Hilbert space for the system under consideration is infinite-dimensional. Therefore, in order to be able to perform numerical computations, it is necessary to reduce the basis chosen for the expansion to a finite number of elements. The number of elements in a basis set will be denoted by N. The equation of motion (12) is then reduced to a set of N×N-coupled differential equations that have been numerically integrated using an explicit Runge–Kutta (4,5) formula. In fact, this is the algorithm used by the most versatile numerical solver available in Matlab [30], which is the computing environment we have been using for the numerical simulations.

The results of the computation will be affected by two sources of error: (1) the numerical integration of the differential equation involving discretization of the time domain, and (2) the reduction in the basis to a finite number of elements. The goodness of a particular choice of basis and of the truncation criterion can be evaluated by comparing the *accuracy* of the results with the *computational cost* required to complete it.

The most important parameter is the number of basis elements. An increase in this number increases the accuracy but also increases the computational cost. However, among all the possible choices corresponding to the same number of elements, there are two important features to look for:

- It is advantageous to choose a basis with some relation to the Hamiltonian of the system so that the basis elements that are discarded by the truncation are the ones corresponding to the higher energies. The energy scale of the system is determined by the equilibrium energies corresponding to $\beta_{H}$ and $\beta_{C}$. The levels whose energy is much higher than the hot equilibrium energy have a low-occupancy probability $P_{n}\to 0^{+}$ as n→+∞. For this reason, the states corresponding to higher energies have a lower impact on the accuracy of the simulation.
- Some bases produce matrix expansions of the operators relevant to the evolution which are mainly composed of empty matrix elements. Such bases carry a smaller computational cost and should therefore be preferred. In order to take advantage of this property, the implementation must resort to sparse matrices.

During the work, we compared two main choices of basis: the *position basis* and the *harmonic basis* (i.e., the set of eigenstates of a harmonic oscillator with a given frequency).

The position basis is the set of eigenstates of the position operator $\hat{Q}$. The corresponding spectrum is continuous and spans the real axis. Therefore, the reduction to N elements involves both a discretization and a truncation to a finite interval [−X,+X]. The differential operators (associated with $\hat{P}$ and ${\hat{P}}^{2}$) can be evaluated with a finite difference method and are represented by matrices whose only non-zero entries lie on the two diagonal bands above and below the main diagonal. The position-dependent operators are represented by diagonal matrices. Moreover, for the potential under consideration, there is a strong relationship between energy and position. States with a lower energy occupy a small portion of the real axis around the origin. Therefore, as long as *X* is sufficiently large, the error due to the truncation is minimal. However, some test simulations suggested that the harmonic basis may be able to achieve better computational performance. The reason for the superior performance is probably the absence of discretization error. Since the harmonic basis is already discrete, the only source of error is due to the truncation. Without the truncation, the equation of motion expressed by Equation (12) over this infinite-dimensional discrete basis would be exact.

The harmonic Hamiltonian whose eigenstates are used as basis is given by

(13) $${\hat{H}}_{B}=\frac{1}{2m}{\hat{P}}^{2}+\frac{1}{2}m\omega_{B}^{2}{\hat{Q}}^{2}$$

**Figure 1:**
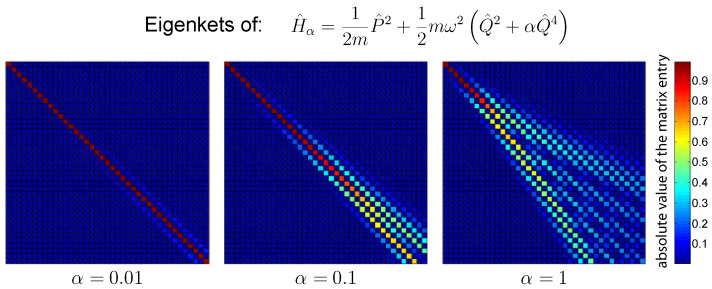
The absolute value of the first 40×40-element block of the change of basis matrix between the Hamiltonian ${\hat{H}}_{\alpha }$ and the harmonic Hamiltonian ${\hat{H}}_{B}$. For these computations, the value of the frequency ω is set to the value of the basis frequency $\omega_{B}$ and is equal to 25. The results are shown for different values of the coefficient α of the quartic term ${\hat{Q}}^{4}$. It is evident that a small value of α, corresponding to a small difference from the harmonic case, results in a change of basis matrix of almost diagonal form. Note that since the vector columns of these matrices are normalized, the maximum possible value of a matrix element is 1.

Figure 1 shows the absolute value of the first 40×40-element block of the change of basis matrix between the normalized eigenstates of the Hamiltonian ${\hat{H}}_{\alpha }$ and the ones of the harmonic Hamiltonian ${\hat{H}}_{B}$ with $\omega_{B}=\omega$. The color corresponds to the absolute value of the matrix element as indicated by the color bar on the right. The three panels correspond to different values of the quartic parameter α. As is clearly visible in the figure, the harmonic basis is characterized by a strong correlation with the eigenstates of the quartic Hamiltonian ${\hat{H}}_{\alpha }$. For a small value of the quartic coefficient α, the change of basis matrix between the two sets of energy eigenstates is almost diagonal, and therefore the error introduced by the truncation is small.

Another important property guaranteed by this choice of basis is that all the operators relevant for the dynamics, i.e., $\hat{Q}$, $\hat{P}$, ${\hat{Q}}^{2}$, ${\hat{P}}^{2}$, ${\hat{Q}}^{4}$ and their linear combinations, exhibit a band structure. To be specific, matrix entries more than 4 elements away from the diagonal are empty. Therefore, the number of operations for the integration of the evolution will increase linearly instead of quadratically with the number of basis elements N. Because of this property, the computational cost of the numerical integration of the equation of motion will be relatively small.

As an initial density operator, we decided to generate a random matrix initially populated only in the first M×M-elements block, with M<N. Moreover, the matrix is built in such a way that the fundamental properties are automatically guaranteed: it has to be a positive-definite Hermitian matrix with a trace equal to 1. As discussed in the first bullet point of this section, the last blocks of the density matrix, which correspond to higher energy levels, have a lower probability of occupation for thermal equilibrium states. Therefore, it would also be possible to represent with high accuracy the density operator corresponding to thermal equilibrium states, as long as the temperature is not too high. However as explained in Section 2.6, when a stable limit cycle exists it does not depend on the initial state. Therefore, the choice of the initial state of $\hat{\rho }$ is irrelevant when the goal is to analyze the behavior of the system at a steady state.

### 3.3. Selection of Harmonic Basis Frequency

We need to select the frequency $\omega_{B}$ corresponding to the harmonic Hamiltonian ${\hat{H}}_{B}$ whose eigenstates are used as the basis. When considering one complete cycle, the frequency ω appearing in the Hamiltonian ${\hat{H}}_{\alpha }$ can assume all values between $\omega_{C}$ and $\omega_{H}$. The values of frequencies outside this interval are not a convenient basis choice, since the resulting eigenstates form a change of basis matrix that has more non-diagonal entries than necessary. This would increase the numerical imprecision introduced by the expansion. However, it is not obvious which frequency $\omega_{B}\in \left[\omega_{C},\omega_{H}\right]$ to choose for the basis Hamiltonian ${\hat{H}}_{B}$. The main options that have been tested are the cold frequency $\omega_{C}$ and the hot frequency $\omega_{H}$ themselves, as well as an intermediate frequency, $\frac{1}{2}\left(\omega_{C}+\omega_{H}\right)$.

**Figure 2:**
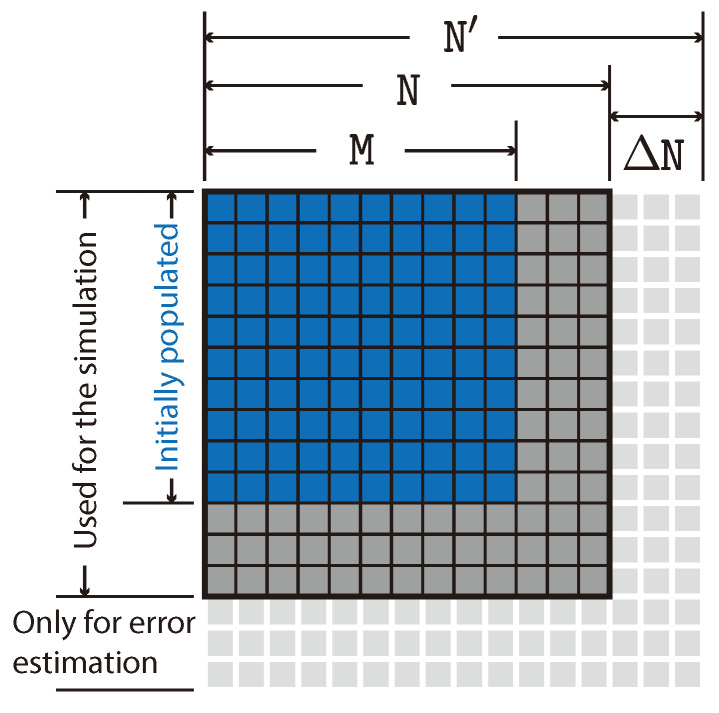
Density operator matrix expansion. The matrix used for the computation is N×N, but initially it is only populated in the first (low-energy) M×M block (blue), with M<N. The augmented $N^{\prime }\times N^{\prime }$, with $N^{\prime }=N+\Delta N$, has only been used to calculate the error estimation quantity ${\dot{\rho }}^{\left(\mathrm{err}\right)}$ defined in Equation (14).

We have developed a method for the estimation of the error introduced by the truncation when the harmonic basis is used. Let the truncated matrix used in the calculation of the evolution be N×N. Part of the calculation is actually performed on a larger matrix, say $N^{\prime }\times N^{\prime }$, where $N^{\prime }=N+\Delta N$. The extra elements of the augmented matrix, i.e., those for which at least one of the indices is greater than N, are used only to compute an error-estimation quantity which we denote by ${\dot{\rho }}^{\left(\mathrm{err}\right)}$,

(14) $${\dot{\rho }}^{\left(\mathrm{err}\right)}=\frac{1}{2\cdot \Delta N\cdot N+\Delta N\cdot \Delta N}\sum_{n,m:\;n\vee m>N}^{N^{\prime }}\left|\frac{d}{dt}\langle n|\hat{\rho }|m\rangle \right|$$

where the denominator of the fraction is the number of those extra elements that are included in the previous summation, i.e., the ones shown as light-gray tiles in Figure 2. The rationale behind this method relies on the fact that the dynamical equations connect only elements of the density matrix not too far from each other. Figure 2 shows a schematic illustration of the density matrix with the initially populated M×M block, the N×N matrix actually used for the computation, and the augmented $N^{\prime }\times N^{\prime }$ used for error estimation.

Suppose that the density matrix is at the beginning of the calculation populated only in the first M×M-elements block,

(15) $$\langle n|\hat{\rho }\left(t\right)|m\rangle =0\;\mathrm{for}\;m\;\mathrm{or}\;n>M$$

As explained in Section 3.2, all the operators involved in the dynamics, once expanded on the harmonic basis, exhibit a band structure. For this reason, when the evolution equation is applied to the density matrix, the resulting matrix will fulfill a similar property,

(16) $$\frac{d}{dt}\langle n|\hat{\rho }\left(t\right)|m\rangle =0\;\mathrm{for}\;m\;\mathrm{or}\;n>M+4$$

The populated block, initially composed of M×M elements, may then grow with the evolution until it reaches the borders of the N×N matrix. It is only then that the truncation starts to perturb the evolution. The quantity ${\dot{\rho }}^{\left(\mathrm{err}\right)}$ can be interpreted as a rate of population flow outside the “main" part of the matrix (i.e., the N×N matrix). This quantity allows us to make some comparisons between the possible choices of the frequency $\omega_{B}$ of the harmonic basis.

**Figure 3:**
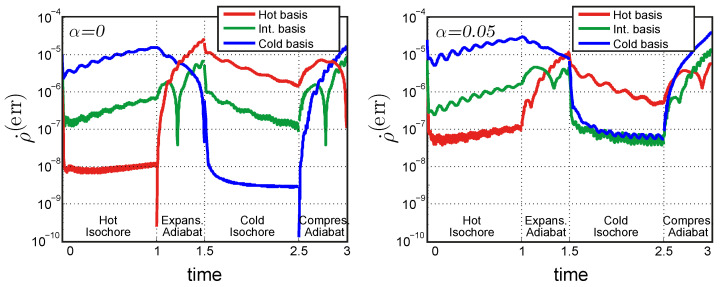
Comparison of different choices of frequency $\omega_{B}$ of the harmonic basis. The amount of error introduced by the truncation is evaluated with the quantity ${\dot{\rho }}^{\left(\mathrm{err}\right)}$. This value can be interpreted as a rate of population flow outside the represented part of the density matrix. It is interesting to notice how the heating (cooling) process increases (decreases) this error rate because a larger (smaller) number of basis elements is required. It is also important to observe that different bases perform better in different parts of the cycle, i.e., whenever the (time-dependent) Hamiltonian frequency is closer to the basis frequency. The right panel shows the non-harmonic case. ${\dot{\rho }}^{\left(\mathrm{err}\right)}$ is slightly larger in this case, but the trend of the curves is the same.

Figure 3 shows the time dependence of this error rate for different cases. The number of basis elements used is always the same, i.e., N=50. The result is pictured after four cycles of evolution from the initial density matrix. In this way it is possible to see what happens when the density matrix is sufficiently near to the limit cycle regime. On the left panel, the Hamiltonian is harmonic, on the right panel it contains a small non-harmonic component, α=0.05. The three different choices of harmonic basis, i.e., $\omega_{B}$ equal to the hot, cold, or intermediate frequency, are plotted in different colors.

The qualitative behavior is similar between the harmonic and non-harmonic cases. The error tends to be larger for the non-harmonic case because of the worst correspondence between the eigenstates of the system Hamiltonian ${\hat{H}}_{\alpha }$ and those of the basis Hamiltonian ${\hat{H}}_{B}$, as shown by Figure 1. The value of ${\dot{\rho }}^{\left(\mathrm{err}\right)}$ is increasing during the hot isochore and decreasing during the cold isochore. This is related to the fact that the heating process increases the energy of the system, which thus requires a larger number of basis elements to be represented correctly. The cooling process has the opposite effect.

Each choice of the basis performs better when the frequency of the Hamiltonian is the same as the one of the basis: the hot basis performs better during the hot isochore, the cold basis during the cold isochore, and the intermediate basis in the middle points of the adiabatic strokes, when the frequency ω(t) is closer to the intermediate frequency $\omega_{B}$.

This observation suggests that the best computational strategy could be to use each of the hot and cold bases on the part of the cycle for which its performance is better and to make a change of basis for the density matrix at two different moments of the cycle: once from the hot basis to the cold one and once from the cold to the hot basis. We tested this approach with a change of basis after each of the adiabatic processes.

The different choices of basis can be compared by analyzing the results and comparing them to a reference solution. For this purpose, we consider the harmonic case. In fact, as explained in Section 3.1, for the harmonic oscillator it is possible to write the evolution equation for the expectation values of a finite set of operators ${\hat{X}}_{k}$ in a closed form, $\frac{d}{dt}\langle {\hat{X}}_{k}\rangle =\sum_{j}a_{kj}\langle {\hat{X}}_{j}\rangle$. This set of coupled equations can always be integrated numerically and the result is very accurate due to the small number of variables involved. The same expectation values can be computed starting from the density operator expansion on the basis sets mentioned above,

(17) $$\langle {\hat{X}}_{k}\rangle =\mathrm{Trace}\left[\hat{\rho }{\hat{X}}_{k}\right]=\sum_{nm}^{N}\langle n|\hat{\rho }\left|m\rangle \langle m\right|{\hat{X}}_{k}|n\rangle$$

The difference between the results obtained with these two methods will be due mainly to the error introduced by the evolution of the expansion of $\hat{\rho }$ on a truncated basis. The error decreases for increasing number N of elements in the basis set. Following the works of Rezek [16,18] on the harmonic quantum engine, we choose the operators $\left\{{\hat{X}}_{k}\right\}=\left\{\hat{H},\hat{L},\hat{D},\hat{1}\right\}$ as basis for the Lie algebra. From the expectation values of these operators, we can compute the expectation values of ${\hat{Q}}^{2}$ and ${\hat{P}}^{2}$. The expectation values computed from the density matrix are denoted by the ρ subscript. As a quantitative measure of the error Δ, the following expression is used

(18) $$\Delta =\frac{1}{2}\left(\frac{|Q_{\rho }^{2}-Q^{2}|}{|Q^{2}|}+\frac{|P_{\rho }^{2}-P^{2}|}{|P^{2}|}\right).$$

Note that, because of the denominators appearing in the definition, Δ can be interpreted as a *relative* error and its value has to be compared to unity. The value of Δ is calculated separately for each of the four strokes of the cycle and then averaged to obtain $\bar{\Delta }$.

**Figure 4:**
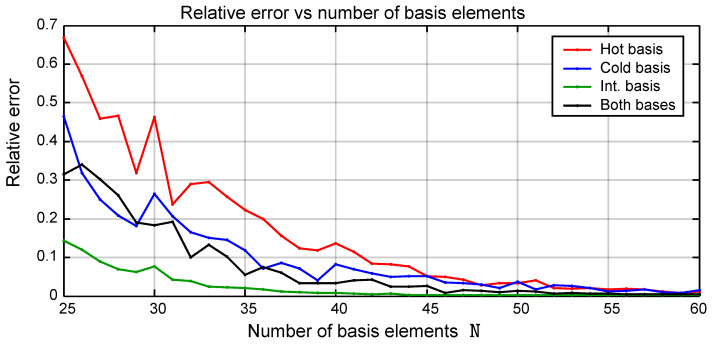
The relative error $\bar{\Delta }$ as a function of the number of basis elements N can be used to compare the performances of the different choices. The different choices of basis are represented with different colors. All the bases give an accurate result if the number of basis elements, N, is sufficiently large. However, the one that shows the best performance is the intermediate frequency basis.

Figure 4 shows the plot of the value of $\bar{\Delta }$ as a function of the number N of basis elements used. The different choices of basis are represented with different colors. The method that involves a change of basis between the hot and the cold frequencies is labeled as “Both bases”. The results corresponding to the position basis are not shown in the figure. The performance of this basis is significantly worse than that of the other choices. Moreover, apart from having to choose a value of N, the position basis involves the additional difficulty of selecting the size of the interval [−X,+X] spanned by the grid points. It must be stressed that this plot can be used only as an indication and does not constitute a precise limit for the error since the result is dependent on the particular choice of the different parameters used for the computation.

All the bases give an accurate result if the number of basis elements is sufficiently large. The intermediate frequency basis seems to perform better than the others and therefore has been used extensively. The performance of the “Both Bases” method is superior during all the four processes of the cycle individually. However, the additional error introduced during the basis change is greater than the advantage, making its performance overall worse than that of the intermediate basis. Section 4 discusses in more detail the error introduced by the change of basis.

The number N of basis elements which is required to reach a given accuracy depends on the physical parameters of the model such as frequencies and temperatures. The following criterion can be used to determine the value of N: a threshold value ${\bar{\Delta }}^{\max}$ is decided for the relative error $\bar{\Delta }$ and the smallest value of N for which the error is smaller than the threshold value $\bar{\Delta }<{\bar{\Delta }}^{\max}$ is chosen. For the parameters used in the simulation of Figure 4, N=60 elements is enough to stay below a maximum relative error of $10^{-2}$ which is a good compromise between precision and computational speed.

### 3.4. Properties of the Density Matrix

It must be checked that the fundamental properties of the density operator are satisfied. For each of the three fundamental properties, a quantity to measure the violation of that property has been defined. The values of these control quantities can be evaluated from the expansion of the operator on a basis. Clearly, since we are numerically integrating the equations of motion and we are truncating our basis to a finite number of elements, we expect numerical approximations to be present in our results. Despite the fact that the governing equations formally guarantee that the fundamental properties of the density operator are preserved throughout the evolution, in reality, we expect to observe a small violation. As long as this is small, it will not affect the reliability of the results.

- The trace of $\hat{\rho }$ is equal to one: $\mathrm{Trace}\left[\hat{\rho }\right]=1$. A violation of this property occurs when the number of basis elements is too small to represent the evolving density operator.
- $\hat{\rho }$ is a Hermitian operator: $\hat{\rho }={\hat{\rho }}^{†}$. The difference between the density matrix and its Hermitian conjugate has been checked to be almost zero for all the tests.
- $\hat{\rho }$ is a positive-semidefinite operator and thus all the eigenvalues $P_{n}$ are non-negative: $P_{n}\geq 0\;\;\forall \;n$. The fraction of negative eigenvalues is a measure of the violation of this property. The initial randomly generated density matrix fulfills this property (as well as all the others). However, we noticed that in the first steps of the simulation, it may depart slightly from the correct value. The evolution will restore permanently the correct values after a few more steps of the computation. The same phenomenon is observed immediately after a change of basis.

### 3.5. Limit Cycle: Convergence and Uniqueness

As discussed in Ref. [31], a system whose underlying Hilbert space is infinite-dimensional is not guaranteed to ever converge to a limit cycle. For some choices of parameters, the internal energy of the ensemble increases cycle after cycle instead of reaching a periodic behavior. The criterion used to establish whether the system has converged to a limit cycle is based on the element-by-element difference between the density matrix at the initial instant of a cycle and at the final instant, denoted, respectively, with the ^(*i*)^ and ^(*f*)^ superscripts. It is quantified as Δ(ρ) defined as

(19) $$\Delta \left(\rho \right)=\frac{\sqrt{\sum_{nm}^{N}\left|\langle n\right|{\hat{\rho }}^{\left(f\right)}|m\rangle -\langle n|{\hat{\rho }}^{\left(i\right)}{\left|m\rangle \right|}^{2}}}{\sqrt{\sum_{nm}^{N}\left|\langle n\right|{\hat{\rho }}^{\left(i\right)}{\left|m\rangle \right|}^{2}}}$$

If the limit cycle is attractive, Δ(ρ) is expected to converge to 0 with increasing number of cycles. Because of the denominator, Δ(ρ) is a relative difference. The difference Δ(ρ) is computed cycle after cycle until it is lower than a certain threshold. This could be, e.g., $10^{-2}$ or $10^{-3}$ depending on the required precision. When Δ(ρ) reaches the prescribed threshold, the evolution is carried on for one more cycle and all the necessary quantities can be extracted from the value of the density matrix at every instant of this last cycle. All the observables computed in this way exhibit a periodic behavior.

Some tests have been conducted in order to check the *uniqueness* of the limit cycle. The same set of parameters, including the set of allocated times on the four strokes of the cycle, is used to compute the evolution of *S* different randomly generated initial density matrices $\langle n|{\hat{\rho }}^{\left(s\right)}|m\rangle$ with s=1…S. In order to verify that the different initial states converge to the same common limit cycle, a measure of the dispersion between the different density matrices is necessary. The quantity σ(ρ) has been evaluated for this purpose,

(20) $$\sigma \left(\rho \right)=\frac{\sum_{s}^{S}\sum_{nm}^{N}\left|\langle n\right|{\hat{\rho }}^{\left(s\right)}|m\rangle -\langle n|\hat{\bar{\rho }}\left|m\rangle \right|}{\sum_{nm}^{N}\left|\langle n\right|\hat{\bar{\rho }}\left|m\rangle \right|}\;\;\mathrm{where}\;\langle n|\hat{\bar{\rho }}|m\rangle =\frac{1}{S}\sum_{s=1}^{S}\langle n|{\hat{\rho }}^{\left(s\right)}|m\rangle$$

Because of the denominator, σ, like Δ, can be interpreted as a relative dispersion. If the different initial states converge to a common limit, the value of σ(ρ) is expected to converge to 0. Even though for some choices of parameters a limit cycle does not exist, we never observed the occurrence of multiple distinct limit cycles for a given set of parameters, i.e., violating the uniqueness property. The relative dispersion σ(ρ) converged to 0 in all cases where a limit cycle has been found.

### 3.6. Non-Linear Coupling with the Heat Bath

The energy levels of the harmonic oscillator are equally spaced by Δϵ=ћω. For this reason, it is possible to represent all transitions between adjacent energy levels with just two operators, the harmonic creation ${\hat{a}}^{†}$ and annihilation $\hat{a}$ operators. The situation for a more general system, such as the oscillator with a quartic term, is very different. The infinitely many energy levels are not equally spaced and an exact treatment of all the transitions between adjacent energy levels would require an infinite number of pairs of ladder operators, $\hat{a}{↓}_{n}^{n+1}$ and $\hat{a}{↑}_{n}^{n+1}$. Analogously, it would also be possible to include transition channels between non-adjacent energy levels.

Some authors, e.g., [32,33], make use of the two ladder operators from the harmonic case also for weak anharmonicity. Since the harmonic ladder operators depend linearly on $\hat{Q}$, this is called *linear coupling of the system with the heat bath*. In different papers, such as [26,27], it is suggested that when the potential is not harmonic, a single pair of approximate ladder operators, ${\hat{a}}_{\phi }$ and ${\hat{a}}_{\phi }^{†}$, may be constructed by letting the coupling contain a more general, non-linear, function ϕ of $\hat{Q}$:

(21) $$\hat{a}=\frac{1}{\sqrt{2}}\left(\left(\frac{\sqrt{m\omega }}{\sqrt{ћ}}\right)\hat{Q}+i\left(\frac{1}{\sqrt{m\omega ћ}}\right)\hat{P}\right)\;\to \;{\hat{a}}_{\phi }=\frac{1}{\sqrt{2}}\left(\left(\frac{\sqrt{m\omega }}{\sqrt{ћ}}\right)\phi \left(\hat{Q}\right)+i\left(\frac{1}{\sqrt{m\omega ћ}}\right)\hat{P}\right)$$

The function ϕ can be computed by requiring that the Hamiltonian $\hat{H}$ satisfy a relation analogous to the one between the harmonic Hamiltonian and the harmonic ladder operators,

(22) $$\hat{H}=\Delta \epsilon \left({\hat{a}}_{\phi }^{†}{\hat{a}}_{\phi }\right)+\epsilon_{0}$$

where $\epsilon_{0}$ is the ground state energy and Δϵ is a suitable factor with the dimension of energy.

As can be seen in [26], in order to compute the approximate ladder operators, it is first necessary to compute the ground state wave function. Let $\psi_{n}\left(x\right)=\langle x|n\rangle$ denote the position expansion of the $n^{\mathrm{th}}$ eigenstate of the Hamiltonian. Since the wave functions cannot be derived analytically for the quartic case, $\psi_{0}\left(x\right)$ must be computed by numerical means. Once $\psi_{0}\left(x\right)$ is known, the generalized coupling function ϕ is defined by

(23) $$\phi \left(x\right)=-\frac{1}{\omega m}\frac{1}{\psi_{0}\left(x\right)}\;\frac{d}{dx}\psi_{0}\left(x\right)$$

Now, it would be straightforward to build the matrix expansion of ${\hat{a}}_{\phi }$ and ${\hat{a}}_{\phi }^{†}$ on the (discretized) position basis since the operator $\phi (\hat{Q})$ would be diagonal and its diagonal matrix entries, $\phi_{k}=\phi \left(x_{k}\right)$, could be computed numerically. However, the harmonic basis has been found to perform better, and building the approximate ladder operators on this basis is more effectively conducted with a different approach. Therefore, we have decided to extract the coefficients, $c_{n}$, of the polynomial expansion of $\phi \left(x\right)=\sum_{n=1}^{p}c_{n}\;x^{n}$, up to the term $c_{p}\;x^{p}$, and use these coefficients to build the approximate operators in the harmonic basis, starting with the matrix expansion of the operators $\left\{\hat{Q},{\hat{Q}}^{2},\ldots ,{\hat{Q}}^{p}\right\}$ on the harmonic basis.

Since the only new term in the Hamiltonian is the quartic term ${\hat{Q}}^{4}$, a natural choice for the maximum polynomial degree *p* is p=3, and in fact, the coefficients of degree greater than 3 turned out to be approximately null for all the numerical simulations considered. The two following constraints are known in advance, $c_{0}=0$ and $c_{1}=1$, since the coupling should restore the harmonic result near the origin. Moreover, the ground state wave function for the Hamiltonian of Eq. 2 is an even function of *x*. The second-order coefficient $c_{2}$ and all the other even coefficients can thus be shown to be zero from parity arguments, and $c_{2}$ has been numerically verified to always be almost 0. In conclusion, the only effect of the small quartic term is to introduce a small third-order term $c_{3}$.

Replacing the harmonic ladder operators $\hat{a}$ and ${\hat{a}}^{†}$ with the approximate generalized ladder operators ${\hat{a}}_{\phi }$ and ${\hat{a}}_{\phi }^{†}$ does not result in a significant difference in the results of the evolution when the quartic parameter α is small. On the other hand, the computational cost required to complete a simulation is significantly increased because of the larger number of non-null matrix elements in the expansion of the Lindblad operators. In conclusion, depending on the value of α, the use of the harmonic ladder operators may be a sufficiently realistic approximation. The dependence of the coefficient $c_{3}$ on the parameter α could provide an assessment on the validity of this assumption. It should be stressed that the non-harmonic $\alpha {\hat{Q}}^{4}$ term present in $\hat{H}$ still affects the evolution of $\hat{\rho }$ due to the unitary term $L_{H}$.

## 4. Computation of Physical Observables

From the matrix expansion of the density operator on a basis {|n〉}, it is possible to extract any kind of physical information. The computation of the expectation value of an operator from Equation (17) is straightforward: once the operator matrix expansion is known, all that is required is a matrix multiplication and the computation of the trace.

On the other hand, the calculation of the probability distribution over the set of eigenvalues of a given operator $\hat{X}$ requires expanding $\hat{\rho }$ over the set of eigenvectors of $\hat{X}$. This operation is performed by the corresponding change of basis matrix T, the matrix whose columns are the normalized eigenvectors of $\hat{X}$ expanded over the original basis {|n〉}. The eigenvectors can be computed using different algorithms. We decided to use a routine based on the Schur decomposition algorithm [34].

It is important to remember that the algorithm is applied to a *truncated* expansion of the operator $\hat{X}$ and therefore the result will also be affected by boundary effects. The accuracy of the result thus depends on the number N of basis elements. Naturally, it is expected that the matrix entries experiencing the largest truncation error are in the vicinity of the truncation border.

In order to quantify this error, we will consider the change of basis matrix between two harmonic bases of eigenstates of $\hat{H}$ and ${\hat{H}}_{B}$ with frequencies given by ω and $\omega_{B}$, respectively. The goal is to estimate how many basis elements are required to obtain an accurate result for the first 40×40-element block of the change of basis matrix T. For this purpose, we performed the following test: the Hamiltonian $\hat{H}$, expanded with a different number of basis elements (i.e., N=40,60,80), is passed as input to the diagonalization routine which computes T. The result is then truncated to the first M×M-element block with M=40. As long as N is sufficiently large, these computations should give the same result.

**Figure 5:**
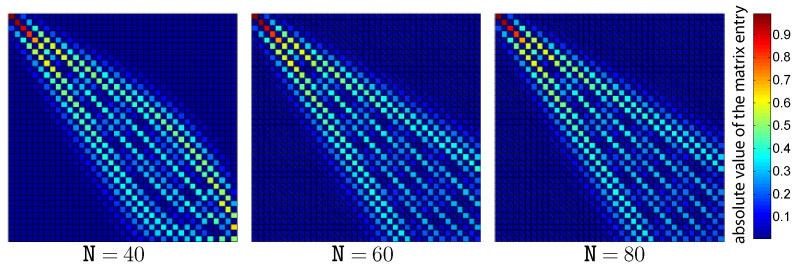
The absolute value of the first 40×40-element block of the matrix of change of basis between two harmonic Hamiltonians with different frequencies. The basis is the set of eigenvectors of the Hamiltonian ${\hat{H}}_{B}$ with frequency $\omega_{B}=15$. The frequency ω of the Hamiltonian $\hat{H}$ is equal to 25. The expansion of $\hat{H}$ over an N-elements basis has been passed as input to a diagonalization routine. The three panels correspond to N=40,60,80, respectively. The result for the case N=40, i.e., the case for which the whole matrix is shown, appears at a glance to be different from the other two cases, meaning that a number of basis elements larger than 40 is required.

Figure 5 shows the absolute value of the entries on the first 40×40-element block of the change of basis matrix T between two harmonic Hamiltonians with frequencies ω=25 and $\omega_{B}=15$. The three panels correspond to a different number of elements of the input matrix, i.e., N=40,60,80, respectively. One observes that the case N=40 gives a result that is clearly different from the other two in the lower right corner (the boundary region) and is therefore incorrect. The computation with 60 elements gives a result that seems completely identical to the one with 80 elements. In this case, 60 basis elements are enough to obtain an accurate result for the first 40 elements.

This example shows that whenever the Hamiltonian operator expansion is truncated, the high-energy eigenstates are the ones affected by the largest error. In order to obtain the correct M×M matrix of change of basis with this method, it is necessary to initially perform the computation using a larger number of elements N>M, and subsequently truncate the result to M elements.

Using this technique, it is possible to compute the probability distribution over the set of eigenvalues of an arbitrary operator as well as the eigenvalues themselves. For example, the computation of the probability distribution $\left\{P_{n}\right\}$ over the set of eigenvalues $\left\{\epsilon_{n}\right\}$ of the time-dependent Hamiltonian ${\hat{H}}_{\alpha }$ allows us to separate the *frictional* and *external* contributions to the total power $P_{\mathrm{tot}}$ exchanged during an adiabatic process as

(24) $$P_{\mathrm{tot}}\left(t\right)=\frac{d}{dt}\sum_{n}P_{n}\left(t\right)\epsilon_{n}\left(t\right)=\underset{\mathrm{Frictional}}{\underbrace{\sum_{n}{\dot{P}}_{n}\left(t\right)\epsilon_{n}\left(t\right)}}+\underset{\mathrm{External}}{\underbrace{\sum_{n}P_{n}\left(t\right){\dot{\epsilon }}_{n}\left(t\right)}}$$

We notice that the frictional contribution to the power has exactly the same expression as the heat exchange rate. In fact, during the isochoric evolution, the energy levels are constant and the first term on the right-hand side of Equation (24) is the only contribution to the energy exchange. It is interpreted as heat transfer. For further details on this distinction see [35] or [19].

The probability distribution $\left\{P_{n}\right\}$ is also necessary for the computation of the energy entropy, $S_{\hat{H}}=-\sum_{n}P_{n}\log\left(P_{n}\right)$. The same can be conducted for the entropy related to any operator. It is interesting to notice that the computation of the energy entropy allows us to deduce also the value of the internal temperature $\beta_{\mathrm{int}}$ that can be generalized to states that are not in thermal equilibrium according to the expression

(25) $$\beta_{\mathrm{int}}=\left(\frac{\partial S_{\hat{H}}}{\partial \langle \hat{H}\rangle }\right).$$

This expression is relevant during an isochoric process for which the Hamiltonian does not depend explicitly on the time.

Finally, the diagonalization of the density operator itself can be useful as well. Denoting by $\left\{p_{n}\right\}$ the set of eigenvalues of $\hat{\rho }$ and by $|p_{n}\rangle$ the corresponding eigenvectors, we can express $\hat{\rho }$ in diagonal form,

(26) $$\hat{\rho }=\sum_{n}p_{n}|p_{n}\rangle \langle p_{n}|$$

From the eigenvalues, it is possible to check whether the positivity property is preserved through the evolution and also to compute the von Neumann entropy $S_{\mathrm{vN}}$ according to the formula

(27) $$S_{\mathrm{vN}}\left[\hat{\rho }\right]=-\mathrm{Trace}\left[\hat{\rho }\log\left(\hat{\rho }\right)\right]=-\sum_{k}p_{k}\log\left(p_{k}\right)$$

where the last equality derives from the general property

(28) $$\log\left(\sum_{n}p_{n}|p_{n}\rangle \langle p_{n}|\right)=\sum_{n}\log\left(p_{n}\right)|p_{n}\rangle \langle p_{n}|$$

which is verified because the operator $\sum_{n}p_{n}|p_{n}\rangle \langle p_{n}|$ is in its diagonal form. For the actual computation of the von Neumann entropy, we had to be prepared that a slight violation of the positivity condition may occur because of the numerical approximations. Some of the eigenvalues of the density matrix may become slightly negative during the evolution. Hence, we discarded these unphysical eigenvalues before applying Equation (27). All the tests performed to check the reliability of these methods produced results that are consistent with the physical predictions. For example, the comparison between the evolution of $S_{\mathrm{vN}}$ and the evolution of $S_{\hat{H}}$ showed all the expected physical properties.In fact, $S_{\mathrm{vN}}\leq S_{\hat{H}}\;\forall t$, whereas the difference between $S_{\mathrm{vN}}$ and $S_{\hat{H}}$ decreases during the isochoric processes, $S_{\mathrm{vN}}$ decreases during the cold isochore, increases during the hot isochore, and is constant during the adiabatic process. Additional details can be found in Ref. [16].

## 5. Conclusions

We approached the problem of how to generalize the study of a quantum heat engine to an ensemble of systems subject to a *non-harmonic* potential. As a model for non-harmonicity, we considered a Hamiltonian that includes a small quartic term. When a quartic term is introduced, the analytical methods usually employed for the harmonic case are no longer applicable and the problem becomes considerably more difficult from a computational point of view.

For this reason, we developed a different and more general approach. We chose to expand the density operator of the ensemble on an appropriate basis and numerically integrate the resulting equations of motion for its matrix entries. Since the dimension of the Hilbert space under consideration is infinite, the basis must be truncated to a finite number of basis elements. The performance of the method chosen must be evaluated by considering both the precision of the results and the computational cost. The choice of basis is critical for both these factors. We analyzed different choices of basis and characterized their computational performance.

For this purpose, we quantified the error associated with the expansion of the density matrix over a truncated basis, we compared the results of our approach for the special case of a harmonic potential with those obtained with the Heisenberg method, and we developed a method to avoid truncation errors when calculating probability distributions over the spectra of physical observables. The density matrix evolution approach has thus proven to be able to produce accurate results and can be used to extract the value of any desired physical quantity since the density matrix carries all the available information about the ensemble. Moreover, it is a very general method and it can also be employed for analyzing systems governed by potentials having a different (i.e., non-quartic) position dependence. For this purpose, the choice of basis has to be carefully studied for the specific potential under consideration in order to obtain the best balance between accuracy and computational cost. In fact, whereas the position basis could seem like the most obvious choice, the error introduced by the discretization creates additional computational challenges that can be avoided by selecting a basis that is already discrete. By choosing the basis of eigenstates of a harmonic Hamiltonian, we are able to easily construct all the operators required to perform the time evolution. As we demonstrate in the present work, rather than using the frequency corresponding to the hot or the cold branches, the most computationally efficient choice is to use an intermediate frequency.

The main limitation of the method is that the integration of the equations is computationally demanding because of the large number of variables involved. The same problem could be addressed with different techniques [36]. A possible candidate is the quantum jump approach, also called the stochastic wave function method or Monte Carlo wave function method. This method consists of computing the unitary evolution governing individual wave functions instead of evolving the density matrix. The dissipative mechanism is simulated as a random process involving instantaneous transitions between states having different energies. However, the quantum jump approach presents its own limitations. In fact, being a Monte Carlo-type method, its solution will always be affected by sampling noise, whereas it can never be completely eliminated, reducing it requires increasing the number of seeds used in the simulation, thus actually limiting the computational efficiency of the quantum jump approach as well.

It is worth emphasizing that simulating quantum heat engines involves unique challenges when compared to (a) *open* quantum systems with a time-independent Hamiltonian and (b) closed systems with a time-*dependent* Hamiltonian. In fact, quantum heat engines involve both mechanisms. For case (a) the eigenvectors of the Hamiltonian provide a natural choice of basis. For case (b) the equations of motion are unitary and the probabilities $p_{n}$ appearing in Equation (26) are constant. Each pure state $|p_{n}\rangle$ in the ensemble evolves independently. Even though in an Otto cycle the time variation of $\hat{H}$ and the non-unitary evolution occur on separate branches of the thermodynamic cycle, neither of the two convenient properties mentioned above is satisfied throughout the complete cycle. Therefore, treating the case of a heat engine requires a special approach to balancing the non-idealities such as the one discussed in the present work.

Employing the algorithm discussed in this work, we have been able to study the thermodynamic behavior of a heat engine using a non-harmonic working fluid and compare the results with the harmonic case. Such a comparison is the subject of an upcoming paper. The present study describes the numerical techniques used in making the comparison.

## Data Availability

The data presented in this study are available on request from the corresponding author.
